# Treatment Cessation in Patients with Diabetic Maculopathy under Intravitreal Anti-VEGF Therapy Following a Treat-and-Extend Protocol

**DOI:** 10.1016/j.xops.2025.100838

**Published:** 2025-06-02

**Authors:** Lucia Saucedo, Isabel B. Pfister, Christin Schild, Justus G. Garweg

**Affiliations:** 1 Swiss Eye Institute, Rotkreuz, Berner Augenklinik, Bern, Switzerland; 2 Department Ophthalmology, Inselspital, University of Bern, Bern, Switzerland

**Keywords:** Diabetic macular edema, Anti-VEGF treatment, Treatment cessation, Treatment interruption, diabetic retinopathy

## Abstract

**Objective:**

To assess the outcomes of treatment cessation due to disease stability in eyes with diabetic macular edema (DME).

**Design:**

A single-center, retrospective, consecutive case series.

**Subjects:**

Patients with DME who had received their first anti-VEGF treatment between 2012 and 2021, a Snellen best-corrected visual acuity (VA) ≥0.1, and a follow-up of ≥24 months.

**Methods:**

Baseline characteristics, best-corrected VA, OCT biomarkers over time, and injection details were collected from patients' medical records. Treatment interruption was defined as a treatment-free interval of ≥25 weeks after the last injection for any reason. An active decision for treatment interruption due to a stable retinal situation was defined as treatment cessation. Data are presented as mean ± standard deviation.

**Main Outcome Measures:**

Percentage of patients experiencing treatment cessation, time to treatment cessation and to reuptake, and change in best-corrected VA and central retinal thickness.

**Results:**

Beyond 109 eyes treated over ≥24 months, 81 eyes (62 patients) met the inclusion criteria. During a follow-up of 5.5 ± 2.3 (median 5) years, patients received 22.6 ± 14.9 (median 20) intravitreal injections, 7.7 ± 3.0 (8.0) of these in the first year. Fifty-seven eyes (70.4%) experienced ≥1 planned treatment cessation of ≥25 weeks, while 4 eyes experienced an unplanned treatment interruption. Treatment cessation was documented in 53 eyes (65.4%) 65.2 ± 52.4 (median 42) weeks after treatment initiation for 106.2 ± 110.4 (median 54) weeks. The reason for treatment cessation was patient-driven in 1 eye (1.9%; the patient wished to stop treatment against medical advice), physician-driven in 38 eyes (71.7%; stable VA, despite persisting residual retinal fluid in OCT), and OCT-driven in 14 eyes (26.4%; no retinal fluid in OCT). Baseline parameters were comparable between eyes experiencing treatment cessation and those which did not.

**Conclusions:**

Treatment cessation was achieved in 70% of eyes with DME after intensive treatment during the first year. This calls for a discussion about a possible systematic assessment of disease stability by omitting a single injection in eyes with stable residual retinal fluid.

**Financial Disclosure(s):**

Proprietary or commercial disclosure may be found in the Footnotes and Disclosures at the end of this article.

Diabetic retinopathy (DR) is a progressive, potentially blinding disease and a primary cause of visual handicap in working-age individuals between 20 and 65 years.^1^ Diabetic macular edema (DME) is a manifestation of DR occurring in all stages of DR.^2^ The global population with DR is estimated to increase by 55% from 2020 to 2045, and the global number of adults with clinically significant DME will rise by 51.9% to 28.6 million in 2045.^3^

Diabetic macular edema shows some pathophysiological similarities with neovascular age-related macular degeneration (nAMD). VEGF has been recognized as a key driver of increased vascular permeability, inflammation, and neovascularization in both diseases. Consequently, intravitreal anti-VEGF therapies have evolved as first-line therapy in these conditions, as they not only prevent blindness but also improve visual acuity (VA) and central retinal thickness (CRT) in a majority of instances in the long term.^4^^,^^5^ While clear guidelines exist for the initiation and long-term treatment of nAMD, including a loading phase with merely 3 monthly anti-VEGF injections followed by an individualized proactive therapy following a treat-and-extend (T&E) protocol, there is less agreement in DME regarding treatment initiation, the number of injections during the loading phase, the use of pro- or reactive treatment protocols (fixed treatment or T&E compared with pro re nata or as needed), and of treatment alternatives, that is, corticosteroids, as a second-line therapy.^6^, ^7^, ^8^

In a previous study, 32% and 40% of our own patients with DME under a pro re nata and T&E protocol, respectively, achieved treatment intervals of ≥12 weeks after 2 years.^8^ Obviously, the treatment demand decreases over time,^9^, ^10^, ^11^ and 24% of patients treated with ranibizumab for 3 years do not need any further intravitreal anti-VEGF treatment (IVT) to maintain their visual and anatomical gains over 5 years of follow-up.^12^ The longer the treatment intervals, on the other hand, the more likely patients have been found to interrupt treatment or be lost to follow-up compared with patients on a fixed 8-weekly treatment interval.^13^ Generally, treatment adherence seems to be linked to functional stability.^14^ and is generally lower in DME compared with nAMD patients, possibly due to the comorbidity profile and the younger age of diabetics.^13^^,^^15^^,^^16^ Beyond physician–patient communication and durability of effect,^17^^,^^18^ perceived visual performance seems to be key to long-term treatment adherence.^19^^,^^20^

Given that treatment adherence is a major concern in DME, it is surprising that the possibility of treatment cessation due to disease stability has only reluctantly been addressed.^21^ Generally accepted criteria for when and how to consider treatment cessation have not been established. We therefore wished to assess the portion of patients with DME under anti-VEGF therapy and a fluid-tolerant T&E in our cohort reaching disease stability, allowing treatment cessation over time.

## Methods

### Study Design

This single-center, retrospective, consecutive case series includes patients with DME who received their first IVT in the Clinic for Vitreoretinal Diseases, Berner Augenklinik, Bern, Switzerland. Patients included in the study attended the clinic between December 2012 and December 2021, were ≥18 years old at baseline, had a VA ≥0.1 (Snellen 20/200), had a follow-up of ≥24 months, and had a signed general consent for the use of their coded clinical data. Patients were excluded if they had received any other previous intravitreal treatment, <3 anti-VEGF injections in the first year, other diseases affecting the macula (such as age-related macular degeneration or amblyopia), if they had received macular laser photocoagulation or any intraocular surgery within 3 months of treatment initiation, relevant vitreomacular traction, a history of retinal detachment, or a systemic disease requiring therapy with corticosteroids or immunomodulating agents. Local institutional ethics committee approval was obtained (Kantonale Ethikkomission Bern, Ref. 2022-01850), and this study was performed in full compliance with the tenets of the Declaration of Helsinki in its latest version.

### Data Collection

The general health situation, including systemic comorbidities and their treatment, VA, and spectral-domain OCT images were collected for all patients at baseline (defined as the day of the first anti-VEGF injection) and annually until the end of 2023 or loss to follow-up. After manual correction of layer alignment, where needed, spectral-domain OCT images were used to determine central subfield thickness (CST), CRT, and the presence of intraretinal or subretinal fluid. Central retinal thickness and CST were measured using spectral-domain OCT (OCT, Heidelberg Engineering). Central retinal thickness was measured manually at the foveal center from the inner retinal surface to Bruch membrane on a micrometer scale. Central subfield thickness was defined as the average thickness within the central macular subfield of 1 mm of the ETDRS grid. Both CRT and CST values were collected as they provide complementary information. While CRT seems to show a moderate correlation to VA, it is not understood as a qualified predictor of the evolution of diabetic maculopathy and change in VA.^22^^,^^23^

Complete intravitreal injection treatment information (agents and dates) was collected for all patients. Based on a maximal treatment interval of 16 weeks under anti-VEGF and of 24 weeks under dexamethasone implant (Ozurdex) according to our in-house guidelines during the study period, treatment interruption was defined as a treatment-free interval of ≥25 weeks after any injection. At the next routine visit after the last injection before treatment interruption, the reason for not receiving another IVT was recorded and classified as either planned treatment cessation (defined as an active decision to interrupt treatment) or unplanned treatment interruption (for any reason, i.e., comorbidity, missed appointment, etc., if an active decision to stop treatment was not documented). In addition, the reason for treatment cessation was further categorized as patient-driven (patient wishes to cease treatment against medical advice), physician-driven (VA stable independently of the presence of stable persisting fluid in OCT), or OCT-driven (no retinal fluid). If the reason for treatment cessation was not documented, we would not assume that it was by patient wishes. This case would therefore be categorized as physician-driven or OCT-driven depending on the presence/absence of fluid in OCT.

Medical history, including VA, CRT, and CST at baseline, at treatment interruption, and every 6 months after this timepoint, was collected.

### Statistical Analysis

Descriptive statistics as well as subgroup comparisons were applied. The Shapiro–Wilk test revealed that the data were not normally distributed. For subgroup comparison, the Kruskal–Wallis H test was applied. The level of significance was set at *P* < 0.05. Data are presented as mean ± standard deviation, and where applicable, median. For statistical analysis, best-corrected VA was converted to ETDRS letter scores, where a Snellen best-corrected VA of 1.0 is defined as 85 ETDRS letters.^24^ All statistical analyses were performed using the SPSS software package v27 (SPSS, Inc).

## Results

Based on the earlier-mentioned inclusion and exclusion criteria, 81 eyes of 62 patients (23.5% of patients required bilateral therapy) with treatment-naïve DME requiring IVT between 2012 and 2023, and a minimal follow-up of 2 years were recruited into this retrospective cohort analysis. The baseline characteristics of the cohort are displayed in Tables 1 and 2.

**Table 1:** Demographic and Clinical Characteristics of Patients at Baseline

Patients (n = 62)	Baseline
Gender (n, males [%])	37 (59.7)
Age (yrs) (mean ± SD)	66.5 ± 10.6
Diabetes type (n [%])	
DM type 1	3 (4.8)
DM type 2	59 (95.2)
Insulin intake (n [%])	40 (64.5)
Hypertension (n [%])	44 (71.0)
Hyperlipidemia (n [%])	18 (29.0)
HbA1c (mean ± SD)	7.7 ± 1.5
Bilateral involvement (n [%])	19 (23.5)

DM = diabetes mellitus; HbA1c = glycated hemoglobin; SD = standard deviation.

**Table 2:** Clinical Characteristics of Included Eyes at Baseline

Eyes (n = 81)	Baseline
Pseudophakia (n [%])	23 (28.4)
Vitrectomy before baseline n (%)	5 (6.2)
Epiretinal membrane (n)	3
Persistent bleeding (n)	1
Complicated cataract surgery (n)	1
DR severity	
Mild NPDR	4 (4.9)
Moderate NPDR	21 (25.9)
Severe NPDR	43 (53.1)
PDR	13 (16.0)
Treatment at baseline (n [%])	
Aflibercept	47 (58.0)
Ranibizumab	28 (34.6)
Brolucizumab	5 (6.2)
Bevacizumab	1 (1.1)
Steroids in the first year (n [%])	4 (4.9)
Dexamethasone implant during follow-up (n [%])	12 (14.8)

DR = diabetic retinopathy; NPDR = nonproliferative diabetic retinopathy; PDR = proliferative diabetic retinopathy.

Over a mean follow-up of 5.5 ± 2.3 (median 5.0) years, patients had received on average 22.6 ± 14.9 (median 20.0) intravitreal injections (Table 3). During the first 5 years of follow-up, the patients received (mean ± standard deviation [median]) in year 1: 7.7 ± 3.0 (8.0), in year 2: 4.5 ± 3.2 (4.0), in year 3: 3.0 ± 2.8 (3.0), in year 4: 3.2 ± 3.2 (3.0), and in year 5: 2.9 ± 2.7 (3.0) IVTs.

**Table 3:** Follow-Up Time and Average Number of Injections in the Study Cohort

Minimal Follow-Up	2–3 Yrs	3–5 Yrs	5–7 Yrs	7–10 Yrs
Eyes (n)	12	29	21	19
Total number of injections received (mean ± SD)	13.3 ± 5.7	18.1 ± 9.1	26.8 ± 11.7	30.8 ± 22.5

SD = standard deviation.

**Figure 1:**
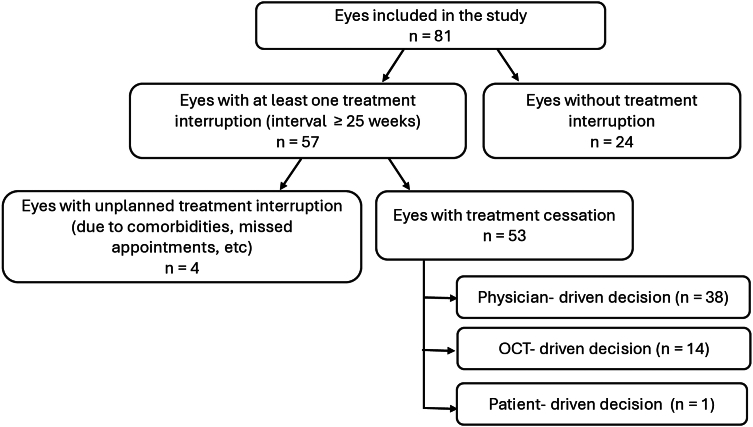
Diagram of the study groups.

Based on the injection dates, we calculated single treatment intervals to identify eyes that met the definition of treatment interruption (treatment-free interval of ≥25 weeks after any injection) at any point during follow-up. In 57 of the 81 eyes (70.4%), a treatment interruption of ≥25 weeks was achieved at least once during the follow-up (Fig 1), and of the 57 eyes with treatment interruption, only 4 eyes experienced an unplanned treatment interruption due to comorbidities or no show ups to appointments. The number of eyes that reached treatment cessation throughout the study follow-up time is presented in Table 4.

**Table 4:** Minimum Follow-Up Timepoints and Eyes Reaching Treatment Cessation at Each Timepoint

Follow-Up	Total	Eyes Reaching Treatment Cessation	
	Number of Eyes (n)	Number of Eyes (n)	Proportion per Year (%)
1 yr	0	28	52.8
2 yrs	9	12	22.6
3 yrs	12	10	18.9
4 yrs	19	2	3.8
5 yrs	13	1	1.9
Total	81	53	100

The proportion is calculated on the total number of eyes of the cohort (n = 81).

Furthermore, there were no differences in baseline parameters between eyes without treatment interruption (n = 24), eyes with an unplanned treatment interruption (n = 4), and eyes with treatment cessation (n = 53; Table 5). Eyes with treatment cessation showed a longer follow-up time and a lower number of injections compared with eyes with treatment interruption (Table S1, available at www.ophthalmologyscience.org) indicating a better therapeutic response, while there were no differences in VA, CRT, or CST between eyes without and with treatment cessation throughout a 4-year follow-up period (Table 6). These results, however, have to be interpreted with care, because the difference in sample size between the 2 groups is large.

**Table 5:** Parameters at Baseline of Eyes with and without Treatment Interruption (Mean ± SD)

	Eyes without Treatment Interruption (n = 24)	Eyes with Unplanned Treatment Interruption (n = 4)	Eyes with Treatment Cessation (n = 53)	*P* Value
VA	69.2 ± 10.4	73.8 ± 4.9	70.4 ± 8.9	0.74
CRT (μm)	474.0 ± 129.7	456.5 ± 95.9	433.9 ± 116.5	0.42
CST (μm)	466.4 ± 103.9	458.0 ± 38.9	436.7 ± 90.7	0.39

CRT = central retinal thickness; CST = central subfield thickness; SD = standard deviation; VA = visual acuity.

**Table 6:** Change of VA, CRT, and CST over Time in Eyes with and without Treatment Cessation (Mean ± SD)

	Eyes without Treatment Interruption (n = 24)	Eyes with Treatment Cessation (n = 53)	*P* Value
VA (ETDRS letter scores) change from baseline to:			
6 mos	5.7 ± 9.2	5.7 ± 9.0	0.79
1 yr	8.3 ± 10.2	7.1 ± 7.6	0.84
2 yrs	6.0 ± 9.7	7.7 ± 7.1	0.37
3 yrs	4.7 ± 8.2	8.7 ± 9.7	0.10
4 yrs	1.1 ± 15.7	6.9 ± 10.2	0.20
CRT (μm) change from baseline to:			
6 mos	−140.1 ± 147.4	−119.0 ± 131.4	0.64
1 yr	−164.4 ± 129.9	−120.6 ± 140.6	0.27
2 yrs	−116.5 ± 157.1	−140.3 ± 131.6	0.61
3 yrs	−101.7 ± 155.7	−146.4 ± 152.5	0.32
4 yrs	−174.3 ± 114.8	−169.0 ± 137.2	0.77
CST (μm) change from baseline to:			
6 mos	−115.9 ± 110.2	−98.4 ± 94.5	0.67
1 yr	−133.6 ± 100.7	−104.8 ± 107.3	0.29
2 yrs	−100.0 ± 111.6	−116.1 ± 95.5	0.47
3 yrs	−99.1 ± 100.1	−120.5 ± 112.1	0.50
4 yrs	−131.6 ± 95.4	−138.4 ± 106.8	0.92

CRT = central retinal thickness; CST = central subfield thickness; SD = standard deviation; VA = visual acuity.

**Figure 2:**
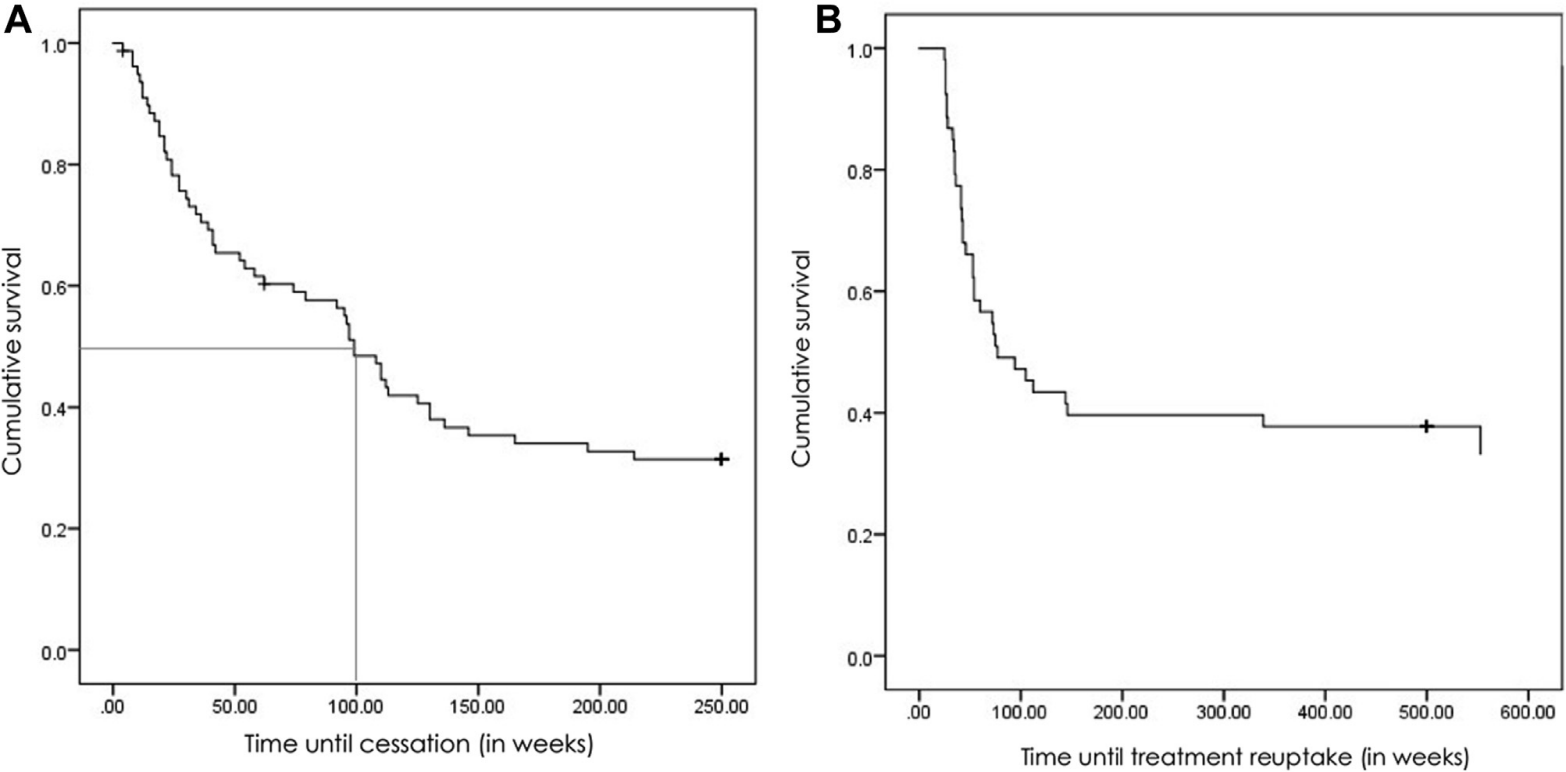
**A,** Kaplan–Meier estimate for the time to treatment cessation. **B,** Kaplan–Meier estimate for the time to treatment reuptake.

With the aim to reduce a potential bias, the 4 eyes with unplanned treatment interruption were excluded from further analysis. Consequently, 53 eyes of the study cohort (65.4%) fulfilled the criteria of treatment cessation, defined as a planned and active decision to interrupt treatment. After (mean ± standard deviation [median]) 65.2 ± 52.4 (42.0) weeks, treatment was paused for 106.2 ± 110.4 (54.0) weeks (Fig 2A, B). The reasons for treatment cessation and the corresponding CRT and CST values at the visit when treatment interruption was decided are displayed in Table 7.

**Table 7:** Reasons for Treatment Cessation and Corresponding CRT and CST Values

	Frequency n (%)	VA (Mean ± SD)	CRT (μm) (Mean ± SD)	CST (μm) (Mean ± SD)
Physician-driven decision	38 (46.9)	77.7 ± 9.3	268.8 ± 58.3	432.1 ± 53.5
1 mm region without fluid	11	78.2 ± 9.2	248.5 ± 38.3	270.4 ± 35.0
1 mm region with residual fluid	27	77.5 ± 9.5	277.1 ± 66.4	315.1 ± 54.8
OCT-driven decision	14 (17.3)	80.4 ± 6.9	248.3 ± 46.9	284.5 ± 32.9
Patient-driven decision	1 (1.2)	-	-	-

CRT = central retinal thickness; CST = central subfield thickness; SD = standard deviation; VA = visual acuity.

Percentage is calculated from the patients who had a treatment cessation (n = 53).

In a subgroup analysis, eyes without retinal fluid at the time of treatment cessation (OCT-driven decision) were compared with eyes with persistent retinal fluid (physician-driven and patient-driven), demonstrating no significant differences in functional outcomes (Table S2, available at www.ophthalmologyscience.org). In addition, we compared eyes based on their CRT at the time of treatment cessation with a discriminatory CRT of ≤320 μm or >320 μm. Again, no relevant differences were identified (Table S3, available at www.ophthalmologyscience.org).

## Discussion

In a European cohort of 81 eyes (62 patients) with DME that have been followed for up to 10 (mean 5.5) years, treatment cessation for a period of ≥25 weeks was reported in 65.4% of eyes 65 weeks after treatment initiation based on function-more than OCT-driven criteria (either physician-driven in the presence of stable VA but tolerance of persisting residual retinal fluid in OCT or OCT-driven in the absence of retinal fluid) without negative impact on the long-term functional and morphological outcomes. This portion is higher than previously reported based on combined functional and OCT-based retreatment criteria: In the open-label extension of the prospective RISE and RIDE studies, continued treatment was not required in ∼25% of eyes in years 4 and 5 if CRT remained <275 μm on OCT and best-corrected VA remained stable (<5 ETDRS letters change versus month 36).^12^ Patients in this study not requiring injections in years 4 and 5 had a shorter duration of diabetes and DME at baseline, were less likely to have proliferative DR, and were more likely to experience DR severity scale improvement of ≥2 steps.^25^ In another prospective randomized clinical trial by the DRCR.net, treatment was paused in years 4 or 5 in ∼50% of eyes after monthly ranibizumab over 36 months if no further improvement was achieved under monthly injections, with resumption of treatment upon worsening.^26^

Treatment cessation in our patients was possible for almost 2 years (106 weeks). This underscores the robustness of stability under our persisting fluid-tolerant treatment protocol,^9^ which goes along with a relevant reduction in the treatment burden for the affected patients. Eyes with DME in this study became widely dry (with tolerance of excess CRT of ≤10%) after an average of 65 weeks, which indicates a generally favorable anatomic response after intensive treatment (mean 7.7 injections) in the first year.^9^ This is well in line with all larger randomized clinical trials,^27^^,^^28^ though with a lower anti-VEGF load. Our results are well in line with the results from the prospective AURIGA study, where patients received monthly visits and 7 aflibercept injections under a pro re nata protocol and achieved a maximal reduction in CRT after the first year, while it remained stable thereafter under ongoing as-needed treatment with a mean of 2 additional injections in year 2.^29^ In an observational study from France using ranibizumab,^30^ patients achieved, after >12 visits and almost 6 injections in the first year, a mean gain of 6 letters despite a CRT of ∼360 um, indicating a significant amount of residual fluid. Given the high treatment burden in this study, it is not surprising that only 63% completed this 2-year study despite a reduced treatment burden in year 2 with 5 visits and 2 injections.^30^ Compared with these 2 studies, the perceived treatment burden may have been remarkably lower in our patients, who enjoyed a medically supported treatment interruption under bi- to 3-monthly visits as long as OCT and function remained widely stable, while the AURIGA and ETOILE patients had to expect an injection at every visit.^29^^,^^30^ While only 5% of our patients were lost to follow-up over a mean of 5.7 years, the prospective phase IV BOREAL-DME study from France also reported—based on similar functional and anatomic responses as in our patients—a sevenfold higher loss to follow-up rate of 35.5% over 36 months,^31^ which is well in line with the 5-year retention rate (68%) in protocol T.^32^ A possible explanation for the difference in treatment adherence and persistence in our patients compared with the aforementioned studies is the integration of the informed patient into the treatment decision.

The anatomic findings in our series are comparable to the above-mentioned studies. These clinical routine studies reported a mean of 2 to 3 injections after year 2 to maintain stability in CRT.^29^, ^30^, ^31^ In randomized clinical trials, >50% of patients did not require an injection in years 4 and 5 based on VA-driven retreatment criteria.^12^^,^^26^ In a post hoc analysis of protocol T, meaningful gains in vision were achieved independent of the presence of persistent DME over 2 years in 44%, 54%, and 68% of eyes receiving aflibercept, ranibizumab, or bevacizumab.^33^ Finally, eyes with stable fluid seem to show a better long-term visual outcome than eyes with larger thickness fluctuation under therapy.^4^ Taken together, stable fluid may be tolerated, which triggers a reconsideration of the best treatment approach. The currently fluid-driven protocol is not necessarily the ideal patient-supported one, as the aforementioned studies show. Eyes with a supportable amount of persistent but stable fluid (in our series CST up to 320 μm) may benefit from an individualized strategy with “diagnostic” treatment interruption.^32^ This strategy clearly differentiates DME from nAMD, where only complete dryness indicates control of disease activity and thus long-term functional stability.^34^, ^35^, ^36^

The robustness of our data is limited by the retrospective nature of this analysis, while the long follow-up contributes to its strengths. Due to the limited cohort size, a possible relationship between the severity of DR and treatment cessation could not be explored. Probably, our findings may not be readily extended to other populations, since Swiss patients have widely unrestricted access to treatment, which may result in improved outcomes compared with other populations with longer times to diagnosis and treatment.^25^ That the portion of patients not reaching a sufficient disease stability in our cohort is comparable to that under treatment with ranibizumab and aflibercept in the DRCR.net protocol T study^37^ indicates at least partial representativity of our findings. Nevertheless, the limited number of cases and the absence of explicit retreatment criteria should trigger a prospective trial systematically confirming our findings.

In summary, a planned treatment cessation was reported in 65% of our patients for almost 2 years after a mean of 65 weeks under therapy. Tolerance of persistent but stable fluid did not result in worse long-term outcomes. A systematic assessment of disease stability by omitting a single injection in eyes with a supportable amount of residual retinal fluid is worth consideration. Further individualization of the long-term treatment may result in an improved treatment adherence of patients with DME.
