# Supplementary material for: Treatment Cessation in Patients with Diabetic Maculopathy under Intravitreal Anti-VEGF Therapy Following a Treat-and-Extend Protocol
Source: Ophthalmol Sci. 2025 Jun 2;5(6):100838. doi: 10.1016/j.xops.2025.100838 (PMC12273413; doi:10.1016/j.xops.2025.100838)
Supplement: Table S1 [file mmc1.pdf]

Supplementary Table 1. Longitudinal parameters of eyes* with and without treatment interruption (mean ± SD)

	Eyes without treatment interruption (n = 24)	Eyes with treatment cessation (n=53)	*p*-value
**Duration of follow up (years)**			
	4.7 ± 2.4	6.0 ± 2.2	0.015
**Number of injections**			
	30.5 ± 17.8	19.4 ± 12.5	0.013
**VA (ETDRS letters)**			
**6 months**	73.9 ± 7.8	76.1 ± 9.1	0.37
**1 year**	77.4 ± 7.4	77.5 ± 7.4	0.84
**2 years**	75.2 ± 10.2	78.2 ± 6.9	0.58
**3 years**	75.4 ± 8.6	78.9 ± 8.1	0.32
**4 years**	71.1 ± 18.0	77.5 ± 8.8	0.20
**CRT (µm)**			
**6 months**	342.7 ± 105.8	312.4 ± 113.5	0.21
**1 year**	309.5 ± 104.2	312.4 ± 125.9	0.46
**2 years**	357.5 ± 139.7	297.1 ± 92.0	0.14
**3 years**	338.0 ± 123.8	282.8 ± 85.6	0.11
**4 years**	288.5 ± 65.4	275.4 ± 72.0	0.60
**CST (µm)**			
**6 months**	357.4 ± 81.7	336.7 ± 87.0	0.38
**1 year**	332.8 ± 70.8	331.9 ± 102.1	0.30
**2 years**	366.4 ± 99.6	321.3 ± 65.2	0.21
**3 years**	342.8 ± 67.7	316.0 ± 60.0	0.23
**4 years**	325.8 ± 45.5	301.5 ± 48.1	0.19

VA = visual acuity, CRT = central retinal thickness, CST = central subfield thickness.
* Four eyes with unplanned treatment interruption were excluded from this analysis (see text in results)
