# Supplementary material for: Treatment Cessation in Patients with Diabetic Maculopathy under Intravitreal Anti-VEGF Therapy Following a Treat-and-Extend Protocol
Source: Ophthalmol Sci. 2025 Jun 2;5(6):100838. doi: 10.1016/j.xops.2025.100838 (PMC12273413; doi:10.1016/j.xops.2025.100838)
Supplement: Table S2 [file mmc2.pdf]

**Supplementary Table 2.** Longitudinal parameters of eyes with or without retinal fluid at treatment cessation.

| | **Patients without retinal fluid (n = 14)** | | | **Patients with retinal fluid (n = 39)** | | |
|---|---|---|---|---|---|---|
| | n | Mean ± SD | Median | n | Mean ± SD | Median |
| **N° of injections before treatment cessation** | | 14.3 ± 8.2 | 14.5 | | 8.9 ± 6.5 | 6.0 |
| **VA (ETDRS letters)** | | | | | | |
| At treatment cessation | 14 | 80.4 ± 6.9 | 82.6 | 39 | 77.9 ± 9.2 | 80.2 |
| 6 Months | 14 | 78.9 ± 5.6 | 77.6 | 39 | 77.5 ± 8.4 | 80.2 |
| 12 Months | 13 | 78.9 ± 5.5 | 80.2 | 38 | 77.2 ± 7.6 | 76.1 |
| 24 Months | 12 | 79.2 ± 7.3 | 80.2 | 34 | 77.1 ± 7.8 | 75.0 |
| **CRT (μm)** | | | | | | |
| At treatment cessation | 14 | 248.3 ± 46.9 | 228.5 | 39 | 268.2 ± 57.7 | 265.0 |
| 6 Months | 14 | 283.4 ± 105.7 | 273.5 | 38 | 295.2 ± 78.0 | 290.5 |
| 12 Months | 13 | 311.8 ± 95.2 | 298.0 | 37 | 293.6 ± 91.4 | 265.0 |
| 24 Months | 12 | 280.1 ± 89.6 | 268.5 | 33 | 281.4 ± 84.1 | 272.0 |
| **CST (μm)** | | | | | | |
| At treatment cessation | 14 | 284.5 ± 32.9 | 278.5 | 39 | 301.7 ± 52.9 | 304.0 |
| 6 Months | 14 | 328.3 ± 75.3 | 315.5 | 38 | 318.6 ± 53.9 | 318.0 |
| 12 Months | 13 | 332.5 ± 64.4 | 341.0 | 37 | 328.4 ± 60.7 | 312.0 |
| 24 Months | 12 | 322.3 ± 53.9 | 317.5 | 33 | 313.0 ± 65.3 | 317.0 |

6, 12 and 24 months timepoints were calculated from the time of treatment cessation. SD = standard deviation, VA = visual acuity, CRT = central retinal thickness, CST = central subfield thickness.
