# Supplementary material for: Treatment Cessation in Patients with Diabetic Maculopathy under Intravitreal Anti-VEGF Therapy Following a Treat-and-Extend Protocol
Source: Ophthalmol Sci. 2025 Jun 2;5(6):100838. doi: 10.1016/j.xops.2025.100838 (PMC12273413; doi:10.1016/j.xops.2025.100838)
Supplement: Table S3 [file mmc3.pdf]

Supplementary Table 3. Comparison of eyes according to a CRT of ≤320 μm or more at treatment cessation (mean ± SD)

	**CRT ≤ 320 μm (n = 47)**	**CRT > 320 μm (n = 6)**
**Number of injections before treatment cessation**	10.5 ± 7.5	8.5 ± 6.0
**Time between baseline and treatment cessation (weeks)**	66.4 ± 50.5	56.3 ± 70.3
**Duration of treatment cessation (weeks)**	102.7 ± 106.4	155.8 ± 150.8
**VA (ETDRS letter scores)**		
At treatment cessation	77.2 ± 8.4	72.5 ± 7.6
6 Months	78.2 ± 8.0	75.0 ± 5.5
12 Months	78.0 ± 7.2	74.8 ± 5.5
24 Months	77.9 ± 7.6	75.0 ± 7.9

SD = standard deviation, VA = visual acuity, ETDRS = Early Treatment Diabetic Retinopathy Study
